# Robot-Assisted Dentistry: What the Evidence Supports and Which Outcomes Are Still Missing

**DOI:** 10.7759/cureus.108601

**Published:** 2026-05-10

**Authors:** Abdulaziz Alghamdi, Ghaith Alqahtani, Almiqdad Dashti, Nawaf Aljameel, Ali Almadluh, Aeshah Agili, Feras Baqazi, Azhar Kutbi, Naza Muhyedin, Fahad Alsaeed, Hanan Al-Qahtani, Noura Alshahrani, Ruba Alshehri, Madawi Al-Qahtani, Abdulaziz Alharbi

**Affiliations:** 1 Faculty of Dentistry, Al Baha University, Al Baha, SAU; 2 College of Dentistry, Imam Abdulrahman Bin Faisal University, Dammam, SAU; 3 Dental Administration, Ministry of Health, Kuwait City, KWT; 4 College of Dentistry, Jazan University, Jazan, SAU; 5 College of Dentistry, Riyadh Elm University, Riyadh, SAU; 6 Faculty of Dentistry, King Abdulaziz University, Jeddah, SAU; 7 Specialized Dental Center, King Fahad General Hospital, Jeddah, SAU; 8 College of Dentistry, King Khalid University, Abha, SAU; 9 College of Dentistry, King Saud University, Riyadh, SAU; 10 Maxillofacial Surgery, Dental Services, King Abdulaziz Medical City, Jeddah, SAU

**Keywords:** ai, artificial intelligence, digital workflow, dynamic navigation, haptic guidance, robot, robotics

## Abstract

Dentistry has rapidly adopted digitally planned workflows, supported by cone-beam computed tomography, intraoral scanning, and virtual planning, expanding computer-assisted implant surgery through static guides, dynamic navigation, and robotic guidance. However, dental robotics is used inconsistently, and conflating robotics with navigation or automation can obscure key mechanistic and safety differences. This narrative review aimed to provide a clinically grounded synthesis of dental robotics across specialties, with emphasis on workflow dependencies, outcome patterns, and translational constraints. A structured literature search was conducted up to March 2026 using PubMed, Embase, Scopus, and Web of Science. Evidence was synthesized thematically, prioritizing clinical outcomes like accuracy, complications, peri-implant parameters, survival, patient- or provider-reported outcomes, workflow data, and implementation considerations. The most mature clinical evidence concerns robot-assisted implant placement, in which high agreement between planned and achieved positions was observed. Yet, accuracy should be interpreted as workflow performance influenced by imaging, planning, registration, tracking stability, and intraoperative conditions. Comparative data suggest the clearest gains versus freehand placement, while early adoption may increase operative time due to added setup and verification steps. Outside implantology, robotics follows distinct trajectories, including robotic orthodontic archwire bending, aligner-related automation, and transoral robotic surgery in maxillofacial practice, underscoring the need to appraise domains using different outcome hierarchies. Restorative and prosthodontic applications are emerging, ranging from robot-guided tooth preparation for crowns, onlays, and veneers to laboratory robotics for manufacturing, yet these remain largely industrial. Across domains, a recurring limitation is the mismatch between commonly reported geometric accuracy endpoints and underreported clinically decisive outcomes, which include complications, patient-reported outcomes, long-term maintenance, and economic value. Cost remains a major barrier, and formal cost-effectiveness evidence is limited.

## Introduction and background

Dentistry has adopted digitally planned workflows, supported by routine use of cone-beam computed tomography (CBCT), intraoral scanning, and virtual treatment planning [1,2]. In implant dentistry, this transition has expanded the use of computer-assisted implant surgery, in which a preoperative plan is transferred to the operative field using static guides, dynamic navigation, or robotic guidance [3]. Shared-control robotics refers to systems in which the clinician retains procedural authority while the device constrains or guides movement within a planned safe corridor. Haptic guidance describes force-based feedback or resistance that helps keep the instrument aligned with the planned trajectory. Active autonomy represents a higher level of robotic execution, in which the system performs a predefined task with less direct manual steering, although clinical supervision and safety oversight remain essential. Dental robotics has emerged within this ecosystem as a precision technology that aims to reduce deviation between intended and achieved treatment, particularly when anatomic safety margins are narrow and prosthetic positioning is demanding [4]. Despite growing interest, terminology remains inconsistent and can blur important mechanistic differences. Navigation systems track the patient and instruments in real time and display positional feedback to the operator, but they do not exert physical control over the handpiece [5]. Automation usually refers to the standardized execution of tasks in laboratory or manufacturing settings, where tasks are repeatable and environmental conditions are controlled [5]. Robotics differs in that it combines sensing, software control, and an actuated component capable of constraining or guiding motion according to a plan [6]. In contemporary dental applications, the prevailing model is shared control, where the clinician retains decision-making responsibility while the system provides bounded guidance.

The most mature clinical evidence for dental robotics is in robot-assisted implant placement. Recent systematic reviews and meta-analyses consistently report high positional agreement between planned and achieved implant positions, commonly expressed as small coronal and apical deviations and low angular error on postoperative assessment [7]. However, these findings should be interpreted as workflow performance rather than actuator performance alone. Accuracy depends on imaging quality, segmentation and planning assumptions, registration fidelity, and tracking stability. It is also influenced by intraoperative conditions that are difficult to standardize, including limited access, saliva and blood, and patient movement. As a result, robot accuracy should be understood as the end product of a chain of interdependent steps, and small errors upstream may propagate despite technically precise motion execution. Comparative clinical research suggests that accuracy gains are clearest when robotic workflows are benchmarked against freehand surgery, whereas contrasts with other guided modalities may be smaller and more indication-dependent. A randomized controlled trial with a six-month follow-up reported improved positional accuracy with robot-assisted placement compared with freehand placement but also longer operative time, reflecting added setup, verification, and recovery steps during early adoption [8]. This pattern highlights a practical trade-off that is central to implementation: precision support may come at the cost of workflow complexity, at least until teams gain experience and processes become standardized.

Outside implantology, dental robotics has developed along distinct trajectories across specialties. In orthodontics, established applications include robotic archwire bending and related fabrication workflows, in which value is primarily linked to manufacturing consistency, standardized wire geometry, and reduced inter-operator variation, rather than to chairside autonomy [9]. In oral and maxillofacial surgery, robotics is most visible through transoral robotic surgery and adjacent applications, where the rationale is improved access, visualization, and instrument articulation in anatomically constrained fields. A recent systematic review summarizing transoral robotic surgery in maxillofacial surgery illustrates both the clinical promise and the dependence on case selection and specialized infrastructure [10]. A recurring limitation across domains is the mismatch between what is easiest to measure and what matters most clinically. The IDEAL framework for surgical robotics provides a structured pathway for development, comparative assessment, and long-term monitoring, with emphasis on learning effects, reproducible reporting, and post-implementation surveillance [11]. Such principles are directly relevant in dentistry because small spatial errors can translate into clinically important consequences and because robotics introduces new failure modes linked to registration, tracking, and workflow interruptions rather than to manual dexterity alone.

The present comparative review aims to provide a clinically grounded synthesis of dental robotics across specialties, with emphasis on mechanisms, workflow dependencies, outcome patterns, and translational constraints. We first clarify definitions and position robotic systems relative to navigation and automation. We then outline the technical foundations that shape performance before synthesizing clinical applications, safety considerations, and practical value propositions across dental domains.

## Review

Search strategy

This review is narrative in nature and does not aim to provide a systematic or exhaustive synthesis of all published studies. Nevertheless, a structured literature search was conducted to identify clinically relevant evidence on dental robotics. From database inception to March 2026, MEDLINE (via PubMed), Embase, Scopus, and Web of Science were searched using combinations of keywords and controlled vocabulary related to dental and surgical robotics (e.g., “dental robotics”, “robot-assisted dentistry”, “robotic implant surgery”, “computer-assisted implant surgery”, “robot-guided implant placement”), as well as adjacent enabling technologies where relevant (e.g., “dynamic navigation”, “surgical navigation”, “haptic guidance”, “tracking”, “registration”). Additional terms were used to capture specialty-specific applications. Reference lists of key articles and recent reviews were screened to identify additional studies. Priority was given to peer-reviewed original research and reviews published in English, with emphasis on studies reporting clinical outcomes (accuracy, complications, peri-implant parameters, survival), patient- or provider-reported outcomes, workflow and learning-curve data, and implementation or economic considerations. Preclinical studies, technical reports, and feasibility studies were included when they informed mechanisms, system design, safety considerations, or translational barriers. The literature was synthesized thematically to integrate findings across study designs and to highlight consistent signals, contextual constraints, and evidence gaps.

Overview of robotics in dentistry

**Figure 1:**
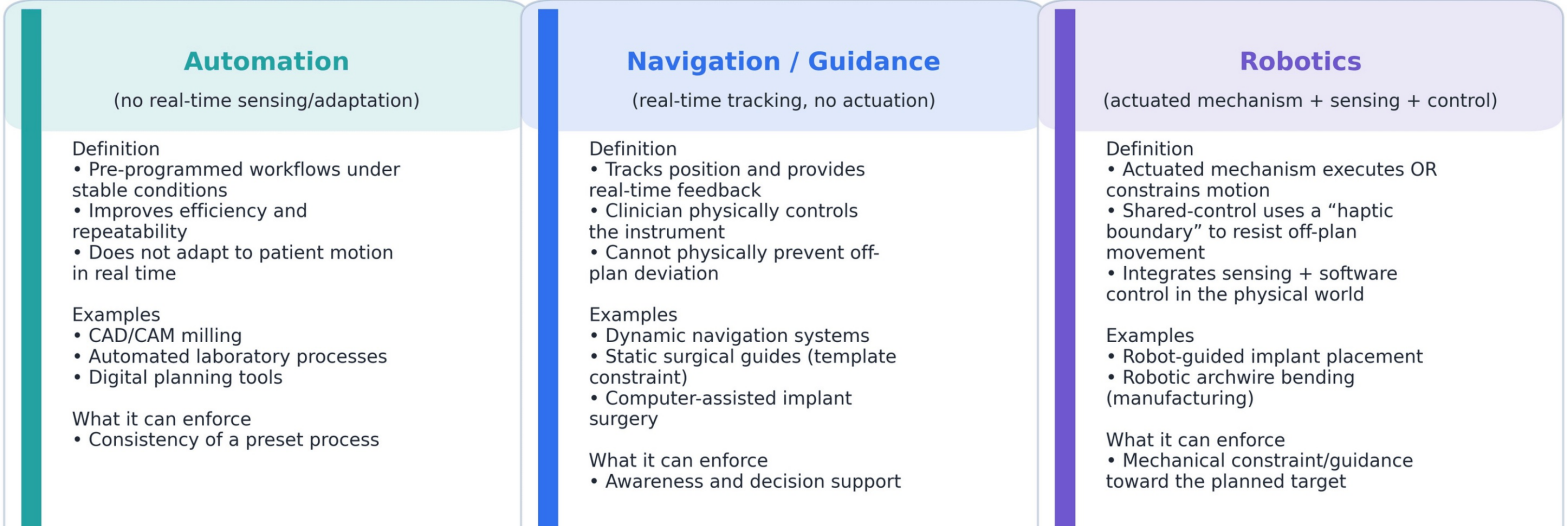
Operational definitions separating automation, navigation guidance, and robotics in dentistry Image Credit: Authors using PowerPoint (Microsoft Corp., Redmond, WA, USA)

The term "dental robotics" is used inconsistently in the literature. A robotic system must do more than provide information. It must include a controllable mechanical component that can execute or constrain motion in the physical world, based on a planned target and real-time inputs. In dentistry, this usually means an actuated arm or tool that interacts with the patient or a dental object, combined with sensing and software control. Implant robots described as semi-active illustrate this concept well. They combine optical navigation with a robotic mechanism that guides drilling and implant placement, rather than only displaying positional feedback [12]. A useful way to separate robotics from adjacent technologies is to focus on who physically controls the instrument and what the system can enforce. Static guides do not qualify as robotics. They are patient-specific templates that mechanically constrain the drill path but do not sense position or adapt during execution. Their strength is simplicity, and their weakness is rigidity. Errors from planning, sleeve tolerance, or seating cannot be detected and corrected by the guide itself. Dynamic navigation sits in a different category. It uses tracking and real-time visual feedback, but the clinician still controls the handpiece freely. It is computer-assisted surgery rather than robotics. This distinction is not semantic. Navigation systems change decision-making and awareness, but they do not physically prevent deviation. In contrast, robot-guided systems can introduce a boundary that resists motion outside the planned trajectory. That boundary is the defining mechanical feature of shared-control robotics. Reviews comparing robot-guided systems with navigation and static guidance often treat them as separate branches under computer-assisted implant surgery, reflecting this functional separation [13]. Within implant dentistry, the clearest clinical examples of robotics are systems built around haptic guidance and real-time tracking. These platforms do not fully automate surgery, but they can constrain the clinician’s movement toward the planned position. This is best described as human-robot collaboration rather than autonomy. The system’s value proposition is not that the robot places the implant. It is the robot's role to help the operator reproduce the plan with fewer uncontrolled deviations while still allowing surgeon-led decisions. Product descriptions for clinical implant robots emphasize haptic guidance and continuous tracking to maintain accuracy even with patient movement, which are core elements of shared-control robotic design. Active robotics is a stronger claim than shared control. It implies that the robot can execute motion with less direct human steering, while the clinician supervises. In the dental implant field, many systems remain closer to shared control than to true autonomy, even when described in ambitious terms. Research reports on implant robots highlight rapid compensation for patient movement through high-frequency monitoring and servo control. This shows genuine robotic actuation and control logic, but it still operates within clinician-planned constraints and safety supervision [14]. Orthodontics provides a second domain where robotics is more clearly defined. Robotic archwire-bending systems physically fabricate individualized wire geometries using programmed motion. Here, the robotic element is not guidance. It is manufacturing. Studies in this area often argue that robotics improves repeatability, reduces wire-bending variability, and supports customization [9]. Engineering reports also describe dedicated robots capable of forming complex multiplanar archwire shapes automatically, reinforcing that this is true robotic actuation applied to orthodontic production tasks [15]. However, much of the orthodontic robotics literature remains dominated by claims of feasibility, technical capability, and workflow. Clinical linkage to patient outcomes is less mature. Many papers infer treatment efficiency benefits without robust comparative clinical endpoints. This leads to an important synthesis point. The same word, robotics, covers two very different evidence ecosystems. Implant robotics is evaluated through surgical accuracy metrics, complication patterns, operative time, and safety margins. Orthodontic robotics is usually evaluated through manufacturing precision, reproducibility, and workflow efficiency. Even when both are robotic, they cannot be appraised using the same outcome hierarchy. Automation is the most common source of confusion. CAD/CAM milling, automated laboratory processes, and digital planning are often presented as robotic dentistry. Yet many of these systems lack real-time sensing and adaptive control. They are automated machines, but not interactive robots in the clinical sense. This difference matters because automation typically improves efficiency and consistency under stable conditions, while robotics introduces additional layers of safety-critical integration with the patient environment. The risk profile changes as soon as a device interacts with living tissue in real time. That is why navigation, guides, and robotics should not be pooled casually in evidence synthesis, even when they are all digital. For this review, a defensible operational definition is therefore needed. Dental robotics should be defined as systems that use actuated mechanisms, guided by software and sensing, to execute or physically constrain clinical or laboratory tasks in dentistry. Static guides and navigation should be treated as enabling technologies, not as robotics themselves, unless they include active mechanical control. This approach aligns with how implant surgery research increasingly categorizes robot-guided, dynamic navigation, and static guidance as distinct modalities rather than a single group (Figure 1). An overview of dental robotic applications across specialties is summarized in Table 1.

**Table 1:** Clinical applications of dental robotics across specialties Synthesized from the literature reviewed in this manuscript [9-15].

Robotics in oral and maxillofacial surgery					
Theme	Core clinical role	Main takeaway	Main advantage	Main limitations and uncertainties	Practical implications
Transoral robotic surgery	Minimally invasive access to anatomically constrained regions of the upper aerodigestive tract and oropharynx	Transoral robotic surgery is the most clinically established robotic application linked to maxillofacial practice, mainly through improved access and visualization rather than automation	Enhanced instrument articulation, stable visualization, and improved ergonomics in deep and narrow fields	High cost, infrastructure burden, and procedure selection constraints; outcomes depend strongly on team expertise and institutional volume	Best suited to centers with established robotic programs and structured case selection; value is driven by access and precision rather than speed
Robotic assistance in reconstructive and microvascular tasks	Support for fine manipulation, suturing, and reconstruction planning in complex surgery	The strongest potential lies in precision support for technically demanding tasks, but routine dental integration remains limited	Tremor reduction and controlled motion for delicate manipulation; improved working comfort in prolonged cases	Evidence is heterogeneous and often procedural rather than outcome-focused; equipment footprint and setup complexity can offset intraoperative gains	Most relevant for high-complexity reconstruction pathways, where precision and surgeon fatigue become limiting factors
Robotic navigation in tumor resection and osteotomies	Plan transfer and intraoperative guidance for bone cuts, margins, and reconstruction alignment	Robotics and navigation are most valuable when millimetric fidelity has direct functional consequences	Reproducible translation of preoperative planning into intraoperative execution; improved alignment of reconstruction segments	Workflow fragility due to registration and tracking demands; limited standardization in reporting and validation	Optimal use requires strict verification checkpoints and clear protocols for conversion when alignment confidence is reduced
Robotics in orthodontics					
Robotic archwire bending	Automated fabrication of individualized archwires from a digital prescription	Robotic wire bending is one of the most mature and practical orthodontic robotics applications because it operates in a controlled manufacturing setting	Standardization of wire form production and reduced dependence on manual bending skills	Clinical benefit is often inferred rather than directly measured; workflow varies between systems and labs	Most useful for practices relying on high-throughput fixed-appliance workflows and consistent wire geometry requirements
Precision, reproducibility, and customization	Repeatable production of complex, multi-plane archwire geometry	The strongest evidence supports improved reproducibility and reduced inter-operator variability in wire shaping	Precise replication of the planned wire form and the ability to produce complex shapes consistently	Precision does not automatically translate into better tooth movement or shorter treatment; biological variability still dominates response	Robotics improves process control, but expectations should focus on consistency rather than guaranteed clinical speed
Integration with digital orthodontics and treatment planning	Linking scans, setups, and appliance design to automated fabrication	Robotics fits naturally within digital orthodontic ecosystems by bridging virtual planning and physical appliance production	Full digital chain from scan to setup to prescription to manufacture	Data quality and planning assumptions can propagate into manufacturing; errors become accurately reproduced	The clinical value is highest when digital planning is already reliable and quality-controlled
Clear aligner workflows	Support roles in aligner production, trimming, finishing, and QA rather than direct chairside autonomy	The contribution of robotics to aligners is primarily industrial automation, not patient-side robotic procedures	High-volume, repeatable tasks where consistency and throughput matter	Often discussed as robotics, despite being automation, clinical outcomes are rarely linked to robotic steps	Best framed as lab/production robotics that improves consistency, not as a chairside clinical robot
Robotics in prosthodontics and restorative dentistry					
Robotic-assisted tooth preparation	Robot-guided preparation of teeth for crowns, onlays, and veneers	This field remains developmental. The concept is attractive, but clinical integration is not yet routine	Highly standardized preparations with clear geometric targets and limited need for intraoperative improvisation	Soft-tissue management, patient movement, and moisture control complicate translation from controlled settings to real chairside practice	Best viewed as an emerging direction rather than a current clinical solution
Margin quality, repeatability, and operator variability	Improving preparation geometry, convergence angles, and margin consistency	The strongest rationale is the reduction of operator-to-operator variability rather than the creation of perfect preparations	Training environments and quality-assurance workflows where repeatability matters	Margin integrity depends on access, tissue condition, and finishing technique; these remain clinician-dependent	Robotics may support standardization, but it cannot replace biological and access-related judgment
Milling, automation, and finishing	Automated manufacturing, handling, polishing, inspection, and workflow orchestration in dental labs	Industrial and laboratory-based rather than chairside	High-throughput tasks with consistent inputs and stable environmental control	Benefits are often operational rather than clinical; outcome gains may not be visible to patients unless quality control improves	Labs can use robotics to improve reproducibility and reduce remakes, especially when linked to consistent digital workflows
Integration with intraoral scanning and restorative workflows	Scan, design, manufacture, and delivery with minimal analog steps	Integration is the key determinant of performance. Robotics adds value when the digital chain is stable and validated	Fully digital workflows with predictable scan quality and consistent design rules	Errors upstream become precisely reproduced, which can increase remakes if QA steps are weak	Clinics should prioritize robust scanning protocols, verification checkpoints, and clear lab communication before adding robotic complexity
Robotics in periodontology					
Scaling and debridement	Robotic or robot-assisted removal of plaque and calculus	The concept is appealing because periodontal instrumentation is repetitive and technique-sensitive, but clinical readiness is low	Standardized surface debridement tasks where motion consistency can be engineered	Subgingival anatomy is variable, and tactile feedback is critical; safety demands are high, and evidence is limited	Not a near-term routine clinical tool; currently best framed as experimental or proof-of-concept
Minimally invasive periodontal procedures	Precision assistance for flapless or minimally invasive access and manipulation	Potential value lies in stabilizing micro-movements and improving access in confined sites	Procedures requiring fine motor control where tremor reduction may matter	Soft-tissue behavior, bleeding, moisture, and limited visualization reduce the feasibility of rigid robotic execution	If translated, adoption would likely occur first in specialized centers and for narrow indications
Standardization of preventive care delivery	Automation of repetitive prophylaxis steps and workflow support	Robotics could support consistency in preventive protocols more than it changes the biological nature of disease prevention	High-volume prophylaxis environments with standardized protocols	Patient variation and comfort limits fixed-motion approaches; benefits may be operational rather than clinical	The most realistic value is quality assurance and protocol consistency rather than improved biological outcomes
Robotics in pediatric dentistry					
Socially assistive robots for anxiety and behavior guidance	Chairside distraction, rapport-building, and behavior support during examinations, restorative treatment, and visits involving local anesthesia	This is the clearest pediatric-dental robotic application with direct clinical evidence. Randomized studies showed improved behavior and lower anxiety-related measures with humanoid robot accompaniment, but the benefit is behavioral/supportive rather than operative	Non-pharmacologic anxiety reduction, better cooperation, and improved child engagement during treatment	Evidence is still narrow, centered on a small number of research groups and social-robot models; outcomes focus more on anxiety/behavior than on treatment efficiency, completion rates, or long-term oral-health benefit.	Best viewed as an adjunct for behavior guidance in selected anxious children, especially when the goal is to improve cooperation without escalating to more invasive behavior-management approaches
Companion robots for children with autism and special healthcare needs	Personalized psychosocial support, familiarization, and co-regulation during specialist dental visits	The most promising niche may be special-care pediatric dentistry. Early dental evidence suggests that companion robots can help some children with autism engage in training and treatment, but the effects are highly individualized and context-dependent rather than universally positive	Predictable, engaging interaction that may ease sensory stress and support desensitization in selected patients	Very limited evidence base, small samples, exploratory designs, and clear reports that robots can sometimes hinder rather than help; effectiveness depends heavily on case selection, timing, and staff integration.	Most appropriate in specialist settings as part of individualized care plans for children with ASD or related needs, not as a standard tool for all pediatric patients
Robot-mediated oral-health education and motivation	Reinforcement of brushing instruction and preventive oral-health messaging for children	In prevention/education, robot-based motivation appears more useful than technically “robotic” treatment. Controlled school-based studies using “The Smiling Robot” found improved plaque outcomes, especially when reinforcement sessions were repeated	High engagement and repeatable delivery of motivational messages in child-friendly formats	Evidence is older, school-based, and preventive rather than chairside clinical; gains may reflect novelty and reinforcement effects, and modern replication is limited	Best framed as a preventive education tool for oral-hygiene promotion, not as evidence of mature clinical robotics in pediatric operative care

Levels of autonomy in dental robotic systems

Across surgical robotics, autonomy is commonly described along a spectrum that includes direct teleoperation, shared control, supervised autonomy, and full autonomy. This spectrum emphasizes how much of the operative task is executed by the system rather than the clinician. It also highlights that autonomy is not a binary state but task-specific, context-dependent, and bounded by safety constraints [6]. When this framework is applied to dentistry, most clinically deployed implant platforms favor shared control over autonomous execution. These systems typically rely on preoperative planning, registration, and real-time tracking. They then constrain or guide the operator’s handpiece movement through mechanical or haptic control. The robotic contribution is therefore best understood as enforced adherence to a planned trajectory, rather than independent surgical decision-making.

The evidence base for implant robotics reflects this design philosophy. Studies and reviews tend to report accuracy metrics and workflow features, but the operator remains central throughout execution [12]. Terms such as “passive,” “semi-active,” and “active” appear across papers, sometimes used to describe comparable shared-control systems. This inconsistency has practical consequences. It limits cross-study comparability and encourages readers to infer different levels of autonomy even when the underlying mechanics are similar. As a result, autonomy categories in dental robotics should be treated as functional descriptions rather than standardized classifications. From an evidence standpoint, greater autonomy should not be equated with better clinical value. Most evaluations remain focused on endpoint deviation and angular error, often under controlled conditions. These outcomes are important, but they are intermediate measures. They do not establish downstream benefits such as fewer complications, improved patient-reported outcomes, or longer-term implant success. Claims of full autonomy in dentistry remain rare and should be interpreted cautiously. A frequently cited example is a case report describing autonomous robotic surgery for an immediately loaded implant-supported full-arch prosthesis. This report is valuable because it demonstrates feasibility and outlines a potential pathway toward greater autonomy in execution. However, its evidentiary weight remains constrained by case-level design and context-specific conditions. It does not resolve generalizability to varied anatomy, restricted access, or unanticipated intraoperative events [16].

Core components of robotic dental workflows

Robotic dentistry is often presented as a single technology. In reality, it is a sequence of tightly linked steps. Each step introduces uncertainty. The final placement accuracy reflects the cumulative error across the chain, rather than the robot alone. This explains why high precision in laboratory tests does not always translate into equally consistent clinical performance. Most robotic computer-assisted implant surgery workflows follow a similar architecture. They begin with imaging and data processing, proceed to virtual planning, and then move through calibration and registration before execution and verification. This stepwise structure is repeatedly described in implant robotics overviews and clinical workflows [17].

Imaging, Segmentation, and Model Generation

The workflow usually starts with CBCT, often combined with intraoral scanning. The purpose is to create a spatial model that can support a planned implant trajectory [18]. Errors introduced here are foundational. CBCT artifacts, segmentation variability, and imperfect surface integration can shift the virtual anatomy away from reality [19]. These upstream deviations are difficult to correct later, because they become the reference for every downstream step. A critical issue is that studies rarely quantify uncertainty at this stage. A more rigorous approach would isolate imaging and segmentation error separately from execution error [20].

Virtual Planning and Intended Targets

Planning is typically prosthetically driven and constrained by bone and anatomic risk structures. Robotic systems do not remove the dependence on planning quality. They amplify it, and if the planned trajectory is suboptimal, the robot may help reproduce the plan accurately, but it cannot rescue the clinical decision [21]. This is one reason why better accuracy should not be equated with better treatment unless the treatment plan is also sound and validated [22].

Calibration of the Robotic System

Calibration links the robotic arm, the tracking sensors, and the virtual plan into a shared coordinate system. Many dental papers briefly mention calibration, but few evaluate it as a determinant of error [13]. Yet calibration defines the relationship between the robot’s internal geometry and the external surgical field [23]. Small calibration deviations can produce consistent systematic offsets that look like clinical drift, even when the operator performs correctly [23]. This step, therefore, deserves greater attention in both reporting and quality assurance.

Registration for the Planned Model

Registration is the most decisive step for clinical accuracy. It maps the real patient to the virtual plan. If the registration is inaccurate, the robot can execute perfectly and still deliver a misplaced implant because it is operating under the wrong coordinate mapping. This is well illustrated by comparative work on registration techniques. Clinical and experimental studies have evaluated marker-based CBCT registration and alternative approaches, showing that the registration method can meaningfully affect performance in robotic workflows [24]. In dynamic navigation, similar concerns arise, with evidence that different registration strategies influence placement-deviation outcomes. These findings generalize to robotics because both modalities rely on the same underlying problem: aligning the planned anatomy with the operative reality. A recurring limitation is that many studies report final deviations without reporting registration quality metrics. This prevents the field from distinguishing whether an inaccurate case failed due to registration, tracking instability, or execution mechanics. Without this separation, conclusions about robotic superiority remain vulnerable to confounding by workflow fidelity.

Real-Time Tracking and Motion Management

Robotic implant systems typically rely on optical tracking and reference markers. This introduces a practical vulnerability. Tracking requires a stable line of sight and stable marker fixation. Clinical environments are dynamic. Occlusion of markers, minor head movement, or changes in retractor position can degrade tracking fidelity. Recent technical and clinical discussions explicitly describe inaccurate registration or marker occlusion as key failure modes in dynamic and robotic computer-assisted implant surgery [25]. Since patient motion is unavoidable, some systems emphasize motion compensation. Engineering and clinical literature increasingly frame motion management as a safety feature rather than a convenience, since even small uncorrected movement can translate into clinically relevant deviation at the apex [26]. This reframes robotic performance as much as an environmental control problem as a mechanical precision problem.

Shared-control constraints and haptic guidance

During execution, many implant robots operate under shared control rather than autonomously drilling. The system constrains the drill to the planned corridor and limits deviation outside safe boundaries. The operator remains responsible for tissue management, irrigation, visibility, and decision-making in response to intraoperative findings. Clinical reports of haptic robotic workflows describe guidance over location, angulation, and depth, which aligns with this shared-control paradigm [27]. This design has an important interpretive consequence. It is worth mentioning that a robot is not a substitute for the clinician. Yet, it is a constraint system that reduces uncontrolled motion. Therefore, differences in outcomes compared with freehand surgery may reflect reduced motor variability rather than improved clinical judgment. Studies that do not address operator skill or learning curves can unintentionally overstate the generalizability of robotic performance.

Verification and Quality Assurance

Verification typically relies on postoperative CBCT and deviation metrics. This is valuable, but it is a late checkpoint. By the time a postoperative deviation is measured, the causal source of error is already hidden within the workflow. A growing body of work aims to standardize procedures and evaluate accuracy under defined protocols, which is a step toward reproducible performance assessment [28]. A stronger standard would include explicit reporting of registration checks, tracking interruptions, recalibration events, and intraoperative stop conditions. Without these data, meta-level conclusions risk comparing clean workflows with real-world workflows rather than comparing devices.

Human-Robot Interaction Models

**Figure 2:**
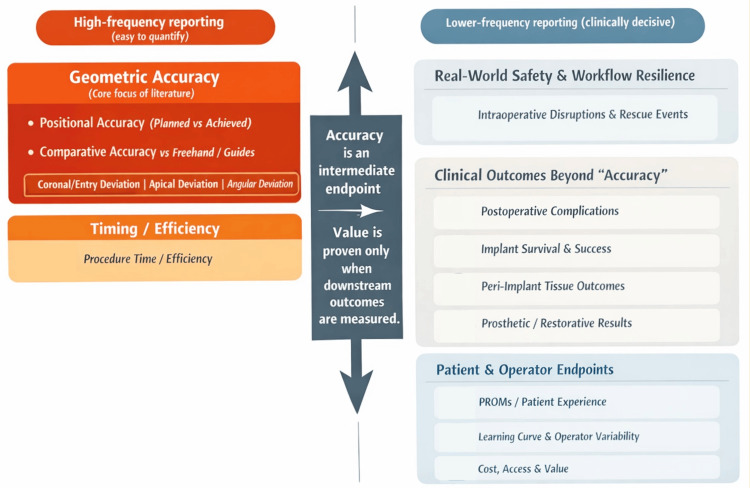
Accuracy and downstream outcome domains in dental robotics Image Credit: Authors using PowerPoint (Microsoft Corp., Redmond, WA, USA)

Human-robot interaction is the functional core of dental robotics. It defines what the clinician does, what the system does, and where control is transferred. In dentistry, this relationship is rarely autonomous. It is primarily collaborative. The clinician remains responsible for decisions, tissue management, and procedural pacing. The robot primarily contributes mechanical constraint, guidance, or precision execution. Most chairside dental robotics systems use a shared-control model. This is most visible in robot-assisted implant placement. The clinician holds the instrument, but the robot shapes the motion through haptic guidance or boundary control. The system does not replace surgical judgment. It reduces unintended deviation from the planned trajectory. Reviews of robot-assisted implant surgery repeatedly describe this approach as haptic feedback combined with real-time tracking rather than independent instrument motion [12]. This interaction model matters because it changes what accuracy means. Visual navigation systems require the operator to interpret a screen and manually translate guidance into hand motion. That translation is cognitively demanding and depends on skill. Haptic robotics shifts part of the control burden from cognition to mechanics. The operator’s movement is physically constrained within a defined corridor. Clinical studies of haptic robotic guidance in implant placement have reported high placement accuracy, including in challenging scenarios such as edentulous cases, which supports the claim that mechanical constraint can reduce motor variability [29]. However, the evidence also shows the boundaries of this benefit. Many clinical reports focus on deviation metrics rather than patient-centered outcomes. The studies are often single-arm or observational. Randomized comparisons remain uncommon. This limits the strength of causal claims about superiority, particularly when outcomes beyond accuracy are inferred. A recent large synthesis of robotic computer-assisted implant surgery highlights high accuracy but emphasizes the need for larger, controlled trials and a better understanding of factors influencing robotic performance [7]. This imbalance in outcome reporting is summarized in Figure 2. Commercial descriptions of implant robots define the system as providing intraoperative robotic navigational guidance rather than executing the procedure independently. A further interaction feature is intraoperative flexibility. Some reports argue that robotic haptic guidance allows plan adjustment during surgery while preserving constraint-based precision. This is clinically relevant because implant planning is often revised based on bone quality, drilling feel, or access. A clinical report describing flapless implant placement with haptic robotics explicitly frames the system as guiding location, angulation, and depth while allowing the operator to modify the plan as needed [27]. The claim is plausible, but the supporting evidence remains narrow. Most studies do not formally assess how often replanning occurs, why it occurs, or whether robotics improves safety during replanning. A different human-robot interaction model emerges in research systems that move beyond shared constraints toward more active execution. Vision-guided robotic proposals and dual-arm strategies are framed as improving positioning and safety through tighter integration of sensing and control [30]. These contributions are important because they outline the pathway toward supervised autonomy. Yet, the clinical literature remains thin. At present, these systems should be interpreted as technical feasibility work rather than mature clinical solutions. Orthodontics illustrates a contrasting interaction model. Robotic archwire bending systems typically operate outside the mouth. The clinician or technician defines the prescription, and the robot manufactures the archwire geometry. The interaction is therefore supervisory and quality-controlled rather than intraoperative. Reviews describe improvements in repeatability and consistency, which are meaningful engineering gains [9]. The clinical translation is less direct. Better manufacturing precision does not automatically translate into better patient outcomes unless it is linked to alignment efficiency, fewer wire adjustments, or improved treatment stability. Across these domains, a consistent limitation appears. Human-robot interaction is often described as a feature, but rarely measured as a determinant of outcomes. Few studies quantify operator workload, trust, overreliance, error recovery behavior, or learning curves as primary endpoints. This is a gap in the evidence base. It also contributes to optimistic narratives that treat robotics as inherently safer, rather than as a system whose safety depends on workflow fidelity, operator competence, and failure management. In dentistry, the most defensible synthesis is therefore that human-robot interaction is currently dominated by shared-control models designed to constrain motion while retaining clinician authority. This design aligns with the realities of chairside procedures. It also provides a realistic boundary for interpretation. Current systems are best understood as precision-support tools, not replacements for clinical decision-making.

Enabling technologies behind dental robotics

Enabling technologies are what make dental robotics clinically credible. A robot can only execute what the system can see, model, and track. For that reason, outcomes depend less on the robotic hardware in isolation and more on the digital infrastructure around it. Imaging defines the anatomy and restorative targets. Planning software translates these inputs into a navigable plan. Registration and tracking link the patient to that plan in real time. Control and feedback modules determine how reliably the system maintains that link during drilling or manipulation.

Imaging and Data Acquisition

CBCT remains the primary dataset for robotic and computer-assisted implant workflows because it provides three-dimensional information on bone volume and proximity to anatomical structures. However, CBCT alone is rarely sufficient for prosthetically driven planning. Surface information from intraoral scans is commonly integrated to refine occlusion, tooth morphology, and restorative contours. This multimodal acquisition strategy is now considered a standard prerequisite for guided implant planning, yet the literature makes clear that the benefit depends on reliable registration between modalities [31]. The fusion of CBCT with intraoral surface scans is not a trivial technical step. It is a major contributor to variability. A study emphasized that accurate registration between these datasets is essential for transferring a virtual plan into a clinically valid guide or navigation workflow [31]. This point is sometimes underappreciated in robotics discussions, where attention shifts quickly to the robotic arm and deviation outcomes. In practice, the robot cannot exceed the fidelity of the spatial model it receives. A recurring limitation across studies is that imaging-related uncertainty is rarely quantified. Many reports treat the planned anatomy as ground truth, then attribute deviation solely to execution accuracy. This is methodologically weak. Segmentation choices, metal artifacts, voxel resolution, and scan-to-scan distortion can each shift the coordinate system before surgery begins. The deviation measured after surgery, therefore, reflects a composite of image error, fusion error, registration error, and mechanical execution error. When these components are not separated, it becomes difficult to interpret why one system appears better than another. Clinical and retrospective comparisons of registration strategies show measurable differences in precision between marker-based and marker-free approaches, suggesting that workflow convenience can be traded off against geometric fidelity [32]. Even when robotics is used instead of navigation, the same principle applies [33]. Accurate motion control does not rescue poor spatial anchoring. Edentulous cases expose the imaging problem most clearly. When teeth are missing, landmark richness decreases, and scan matching becomes less stable. Robotics papers often report strong accuracy in selected indications, but generalization to low-landmark conditions depends on how the workflow secures reference markers and maintains tracking reliability. Technical discussions of implant robotics explicitly note field-of-view constraints and marker visibility challenges in the narrow oral environment, which are directly tied to how imaging references are acquired and maintained [14]. Facial scanning is sometimes proposed as an additional input for virtual patient construction, but its role in robotics remains indirect. The clinical value is strongest when facial data improves esthetic planning and prosthetic orientation. It is less clear that facial scanning improves robotic execution itself, unless it contributes to more robust reference geometry or patient-specific alignment strategies. Much of this remains conceptual, and comparative outcome studies do not yet support it.

Virtual Planning and Digital Workflows

Virtual planning is the stage where robotics becomes clinically meaningful. If planning quality is poor, robotics may reproduce the wrong target with high precision. In contemporary implant workflows, virtual planning is usually built on a merged dataset. CBCT provides bone morphology and risk anatomy. Intraoral scans add occlusal and restorative surfaces. This merged virtual patient is then used for prosthetically driven implant positioning and surgical pathway design. A recent overview of guided implant therapy describes this integration as the standard digital sequence leading to planned placement and transfer to surgery [21]. This planning model often includes a digital wax-up. The wax-up serves as a prosthetic endpoint, determining the number of implants, axis, and emergence profile.

In this sense, the planning step is not only geometric. It is restorative decision-making translated into spatial constraints. CAD/CAM integration serves two roles. The first role is transfer. Planning outputs are translated into physical devices, such as static guides, scan appliances, or reference components. The second role is verification and standardization. Fabricated components can stabilize registration and reduce operator variability. These advantages are repeatedly cited in digital workflow literature. Yet CAD/CAM also introduces new failure modes, such as manufacturing tolerance, sleeve play, guide deformation, and seating errors. These issues are well known in guided surgery. However, they are still inconsistently addressed in robotic discussions, where attention may shift away from the transfer device and toward the robot itself. The evidence base for fully digital workflows is strongest in static guided surgery, where planning-to-transfer accuracy has been examined prospectively. A study evaluated the clinical accuracy of static-guided implant surgery planned using a fully digital protocol, highlighting that digital planning is not merely a convenience but a distinct workflow that requires clinical validation [22]. However, the central endpoints remain measures of deviation. Patient outcomes and complications are analyzed less consistently, and follow-up is often limited. Robotic and dynamic systems mitigate some limitations of static guides, but they do not eliminate the need for planning fidelity. Dynamic navigation is a useful comparator here because it shares planning inputs but differs in transfer method. Prospective clinical evidence shows that both dynamic navigation and static guides can improve accuracy compared with freehand surgery, with smaller differences between navigation and static guidance depending on the metric used [33]. This suggests that planning is a major determinant of performance across modalities and that the transfer mechanism may yield incremental gains rather than a complete step change. Edentulous workflows expose the most important planning constraints. Landmark scarcity increases registration uncertainty.

Diagnostic dentures and scan appliances are often used to introduce reference geometry for prosthetically driven planning. This adds complexity. It also adds opportunities for cumulative error, particularly when multiple scans are required. Recent technical work has sought to streamline these steps, including single-scan approaches that integrate diagnostic denture information into navigation workflows [34]. The existence of these proposals reinforces a key point for robotics: planning is not a neutral prerequisite. It is a performance bottleneck that often determines whether the robotic execution can be trusted. A further limitation is reporting quality. Many articles describe digital planning as if it were standardized, yet software platforms, registration pipelines, and operator checks vary widely across studies. Without consistent reporting of merging methods, calibration steps, and verification procedures, it becomes difficult to compare evidence across systems. This heterogeneity is one reason why conclusions in the robotics literature should be framed cautiously. Current studies support improved geometric precision under digital workflows, but stronger clinical evidence is still needed to link these planning advantages to patient-centered outcomes and long-term treatment success [21].

Tracking and Navigation

Tracking is the operational backbone of navigation and robot-guided dentistry. It determines whether the system knows where the patient is in space, where the instrument is, and how both relate to the planned anatomy. When tracking fails, accuracy becomes irrelevant. The workflow collapses into manual surgery or requires re-registration. This dependency explains why many discussions of robot performance are, in practice, about tracking robustness. Optical tracking is the dominant approach in dental navigation and most robot-guided workflows. It offers stable geometric measurement in typical clinical environments and is not inherently distorted by metal objects. Its weakness is line-of-sight dependency. The oral cavity is small, crowded, and frequently obstructed by hands, retractors, suction, and the handpiece. These conditions increase the likelihood of intermittent marker occlusion. Patient head movement can rapidly degrade positional fidelity if the reference frame is not continuously and reliably tracked. Recent work on robotic and navigated systems highlights that motion management and marker visibility are central safety concerns, rather than minor technical limitations [26].

Electromagnetic (EM) tracking is often presented as a solution to line-of-sight limitations. It can, in principle, maintain tracking when optical markers are hidden. This advantage is meaningful in dentistry, where visibility is frequently compromised. A modern EM implant navigation approach reported that EM tracking could address line-of-light hidden problems and simplify operation procedures in experimental settings [35]. However, EM tracking introduces its own vulnerabilities. Field distortion from conductive materials, equipment, and motors remains a serious concern. Early work in computer-aided implant placement explicitly argued that EM tracking was unsuitable for implant drilling because the drill motor produced substantial magnetic distortion, which impaired visualization and tracking reliability [36]. The contemporary literature suggests technical progress, but it also indicates that EM methods require careful validation under realistic clinical interference conditions, not only under controlled experimental environments [35].

Fiducials and reference markers are the practical interface between tracking theory and chairside performance. Navigation systems typically depend on an intraoral or extraoral reference marker fixed to the patient, with a separate tracker attached to the handpiece. This arrangement allows the software to express instrument motion relative to the planned anatomy. In endodontic dynamic navigation, systematic review evidence clearly describes this configuration. It shows that optical tracking is the prevailing method, with the reference marker serving as the anchor for real-time guidance [37]. The same principle carries over to implant workflows. The distinction is that implant drilling often involves higher forces and greater movement, which can make maintaining marker stability more difficult throughout the procedure. A key limitation in the evidence base is that tracking quality is rarely reported as a measurable variable. Many studies present implant deviations as outcomes without documenting how often tracking was interrupted, whether recalibration was needed, or whether re-registration occurred. This weakens the interpretation because poor outcomes may arise from workflow instability rather than intrinsic limitations of the navigation or robotic platform. Some clinical studies show that dynamic navigation can achieve clinically acceptable accuracy but also demonstrate that changes in workflow can alter results, implying sensitivity to process fidelity [38]. The implication is that tracking technology should not be evaluated in isolation. It must be assessed as part of a full clinical workflow. Comparative syntheses reinforce this point. Recent analyses of computer-assisted implant surgery distinguish static guides, dynamic navigation, and robot-guided systems, but accuracy differences across modalities are often modest and heterogeneous [33]. This heterogeneity likely reflects variation in registration and tracking execution rather than device physics alone. Studies that treat navigation or robotics as a single intervention without characterizing tracking interruptions or marker-stability risk overstate generalizability.

Actuation and End-effectors: Drilling, Cutting, and Manipulation

Actuation is the component that converts a digital plan into physical motion. It is the work-producing element of a dental robot. In most systems, actuation is not a single part. It includes the actuator itself, the transmission pathway, and the control architecture that regulates motion. The dental robotics literature often treats these elements as background engineering. Yet they are central to both accuracy and safety. A recent implant robotics overview explicitly frames the actuation and drive-transmission modules as functionally equivalent to a human hand. At the same time, servo control and software form the brain that regulates movement [14]. In implant robotics, the most established actuation strategy is not fully autonomous drilling. It is shared-control guidance. A well-described example is the haptic paradigm, in which the clinician moves the handpiece while the robotic mechanism constrains the direction and depth. One review describes a mechanical measurement arm connected to the patient, with joint encoders tracking the drill's position and haptic feedback limiting motion outside the planned trajectory [14]. This design makes an important clinical trade-off. It reduces uncontrolled deviation without removing clinician control of access, irrigation, and tactile monitoring. The choice of end-effector is equally consequential. Many haptic-guided systems use standard implant drills as the operative tool. This improves clinical familiarity and allows normal irrigation and visibility compared with bulky guide sleeves. A clinical report on flapless, robot-guided placement highlights the use of standard drills as end effectors within a haptic workflow while maintaining typical intraoperative visibility and cooling [27]. These are practical advantages, not minor conveniences, as limited mouth opening and posterior access are common constraints in implant surgery.

Static guide stacks can increase required drill length, which may worsen access in challenging cases. By contrast, systems described as active robots imply a higher degree of robot-driven motion. This approach changes the physical demands on the end effector and the operator environment. A review notes that when an active robotic arm autonomously enters and exits the oral cavity, procedural time may increase [17]. This observation captures a broader issue. Greater mechanical autonomy can increase complexity at the exact point where dentistry has the least tolerance for obstruction, collision risk, and restricted working volume. The clinical relevance of these design choices is not yet established by evidence from outcomes. Comparative work suggests that differences between active and semi-active designs do not necessarily produce meaningful gains in placement accuracy. A review found no significant differences in accuracy between active and semi-active implant robots, despite the systems differing mechanistically [7]. This cautions against assuming that higher actuation autonomy yields superior clinical performance. In many workflows, registration quality and tracking stability may dominate the error budget, reducing the marginal benefit of more complex actuation. Some emerging systems attempt to extend actuation beyond geometric constraints into adaptive control. Vision-guided proposals include end-effector-mounted cameras and real-time torque monitoring to adjust drilling or tapping based on bone density and patient motion [30]. These features are conceptually attractive because they shift robotics toward closed-loop interaction with tissue. However, most supporting evidence remains technical or early-stage. The key question is not feasibility. It is whether adaptive actuation improves clinically relevant endpoints, such as overheating risk, cortical perforation, or complication rates, under routine conditions. Force and torque sensing are often discussed as future requirements for safe robotic manipulation in confined anatomical spaces. Surgical robotics literature describes haptic platforms as two-way systems, in which tool-tissue forces are detected at the end-effector and relayed back to the operator as feedback [39]. Dentistry is well-suited to benefit from this architecture because tactile cues are central to drilling control and bone quality assessment. Yet current studies on dental implant robotics rarely quantify force control performance. This limits conclusions about whether robotics improves safety beyond improving positional accuracy.

Haptic Guidance, Force Sensing, and Control Stability

Haptic guidance is the defining interaction feature of most chairside dental robots. It converts a virtual plan into a felt constraint. The clinician remains the active agent, but movement is steered by resistive or directional forces that discourage off-plan trajectories. This approach is often described as a robotic analog of static guidance, with the added advantage of real-time adaptation. In implant placement, haptic guidance is often framed as a safety mechanism. It aims to limit unintended angular drift and depth errors, particularly when access is constrained and visibility is imperfect. Preclinical work using haptically operated robotic guidance has reported high placement accuracy and has been extended to clinical demonstration, reinforcing its feasibility under controlled conditions [40].

The more important question, however, is how much of this accuracy is due to haptic constraint itself versus the broader workflow, including registration quality and tracking stability. Most studies do not disentangle these components, which limits interpretation when robotics is presented as the causal driver of precision. Comparative experimental work that separates implant robots by their human-robot interaction designs suggests that interaction mode can influence both efficiency and accuracy, even when the planned task is the same [41]. Force and torque sensing extends robotic support beyond geometry. Instead of only constraining position and angulation, sensing-based systems can observe tool-tissue interaction during drilling and insertion. This has two potential clinical roles. The first is safety: identifying abnormal resistance patterns that may indicate overheating, poor irrigation, or a cortical breach. The second is quality control, which supports the assessment of primary stability. In an implant robotics progress review, force sensing in an autonomous platform was reported to correlate with implant insertion torque in vitro, suggesting that real-time force signals could serve as a surrogate for stability-related endpoints [14]. This is conceptually attractive, but current evidence remains preliminary and largely experimental. Correlation under laboratory conditions does not establish a reliable clinical prediction, particularly when bone quality and drilling dynamics vary across patients. Some newer systems propose torque-sensor-equipped robots capable of real-time control of drilling forces and torques under surgeon supervision, with partial automation of drilling and tapping [30]. These designs reflect an important shift. They move robotics closer to closed-loop control, where the robot adapts motion based on sensed resistance rather than only enforcing a preplanned path. The clinical value of this step remains uncertain because few dental studies link force-controlled actuation to outcomes such as reduced thermal injury, fewer complications, or improved implant stability distributions. Control stability is the hidden determinant of whether haptic and force feedback are clinically usable. A robot can only guide safely if its response is smooth, predictable, and resistant to oscillation. Dentistry is particularly sensitive to instability because the operative field is small and tool-tissue contact is continuous. High stiffness can improve positional fidelity but may transmit uncomfortable forces to the operator and patient. High compliance can feel safer but may reduce reproducibility. These trade-offs are well recognized in broader surgical robotics research, where haptic feedback is described as valuable for trajectory guidance and for avoiding critical regions, yet technically challenging to implement without introducing unwanted dynamics [42]. A further difficulty is the interaction between guidance forces and force-feedback signals. When both are present, one can mask the other. In the surgical robotics literature, studies have shown that superimposing guidance and tactile information can lead to overlap and reduced interpretability, which then affects usability and potentially safety [43]. This issue is directly relevant to dental robotics. If the operator cannot distinguish boundary resistance from tissue resistance, the system may reduce rather than enhance situation awareness. Engineering solutions increasingly focus on improving stability through compliant actuation and better torque control. For example, a recent study on force feedback in teleoperated surgical robotics reported that series elastic actuation reduced spike-like torque error and improved control response behavior [44]. Although this evidence is not dental-specific, it addresses a shared problem: stable force rendering is required if haptics are to be informative rather than disruptive.

Software, Usability, and Workflow Integration

Software is the practical interface of dental robotics. It determines how plans are created, how registration is performed, and how guidance is delivered during surgery. In many clinical reports, the robot is treated as the intervention. In reality, outcomes depend on the full digital workflow and the quality of user interaction with it. A recent review of dynamic and robotic computer-assisted implant surgery highlights that these systems require new operator competencies and a shift in clinical roles, which is largely mediated through software rather than hardware [25]. Most implant robotics platforms rely on software that links imaging, virtual planning, registration, tracking, and intraoperative guidance into a single chain. This chain is sensitive to small procedural deviations. A motion-compensation paper describes workflow integration as technically demanding because it requires precise marker registration, stable tracking, and synchronized equipment behavior; failures at any of these steps can increase operative time and compromise accuracy [26]. The implication is that usability is not a superficial issue. It is a determinant of clinical performance. Planning software is often presented as mature because it resembles guided-surgery planning. Yet robotics places more weight on planning fidelity. The plan is not only a reference. It becomes the geometry that the system tries to enforce. This increases the consequences of segmentation errors, incorrect merging, or unrealistic drill paths.

Many studies report final deviation metrics without reporting how planning uncertainty was handled or checked. That omission weakens causal interpretation. It also limits reproducibility across clinics and software ecosystems. A scoping review assessing outcome measures in computer-assisted and robotic implant surgery underscores heterogeneity in how studies define and report evaluation endpoints, which mirrors broader inconsistency in software-dependent workflows [45]. Registration and calibration are the most user-sensitive steps. They are often described briefly, but they demand structured interaction and verification. When software provides weak feedback on registration quality, operators may proceed with fragile alignment. This can produce high-confidence guidance built on an incorrect spatial map. Evidence from robotic workflows supports the view that standardized operating procedures can improve consistency. Still, the existence of such procedures also suggests that results are workflow-dependent rather than device-intrinsic [28]. Intraoperative interfaces also shape cognitive load. Navigation systems require continuous visual attention and mental translation from a screen to hand movement. Robotic haptic guidance reduces that translation burden by mechanically constraining motion, but software still governs how the operator confirms targets, interprets warnings, and manages tracking interruptions. Current evidence rarely measures these human-factors endpoints directly. As a result, many claims of improved efficiency or ease remain weakly supported by formal usability assessment. Data on the learning curve further illustrate why software design matters. A review of learning curves in robotic and dynamic computer-assisted implant surgery notes that robust evidence of learning curves for robotic systems remains limited and has been a recognized knowledge gap [46]. More recent experimental work has begun to examine learning trajectories in robot-assisted implant placement among trainees. Still, much of this remains in vitro or early-stage, and it cannot substitute for real-world adoption evidence that includes workflow disruptions and case complexity [47]. Training also remains a practical bottleneck. Reviews note steep learning curves and vendor-specific training with limited standardization across platforms [48]. Workflow integration also involves time. Robotics adds steps that are not present in freehand surgery, such as registration, calibration checks, and marker management. These steps may be offset by faster execution during osteotomy and placement, but the balance is procedure- and team-dependent. Independent clinical timing studies remain comparatively scarce. A broader surgical robotics perspective underscores the need to treat usability, training, and systems implementation as essential endpoints rather than optional add-ons. The IDEAL Robotics framework explicitly emphasizes human factors and ergonomics during translation, because poor usability can undermine both safety and adoption even when technical accuracy is high [11]. This aligns closely with dentistry, where the constrained workspace magnifies the effects of minor workflow friction.

Emerging Role of AI In Robotic Planning and Intraoperative Adaptation

AI is increasingly positioned as the intelligence layer that can extend dental robotics beyond mechanical precision. In the current literature, AI contributes mainly in two ways. It supports preoperative planning by automating the interpretation of imaging and proposing plan elements. It also supports intraoperative adaptation by improving tracking, registration, and motion compensation. These contributions are conceptually important, but the clinical evidence is still uneven. In preoperative planning, AI is primarily used for time-consuming, operator-dependent image-processing tasks. This includes segmentation of CBCT volumes, identification of anatomical structures, and extraction of geometric features that guide implant positioning. A recent systematic review of AI in implant dentistry shows rapid growth in this area but also highlights that most work remains developmental rather than clinically validated at scale [49]. Many studies report high performance on curated datasets, yet generalization across scanners, artifacts, and patient populations is rarely tested rigorously. This limits confidence in how AI-supported planning performs under routine clinical variability. The promise of AI planning is often framed in terms of improved accuracy and predictability. This claim requires careful interpretation. Better segmentation or landmark detection can improve the planning model, but this does not automatically improve the final clinical outcome. Clinical performance still depends on registration fidelity and execution stability. Preclinical work on AI-assisted implant planning tends to evaluate precision in virtual environments rather than the full end-to-end chain from imaging through placement [50]. The practical contribution of AI is therefore best understood as reducing upstream uncertainty and operator variability, rather than guaranteeing superior implant positioning. AI is also increasingly integrated into intraoperative systems for navigation and real-time control. Some reviews describe AI-enabled workflows that integrate image processing with dynamic tracking devices and robot-assisted placement, aiming to reduce deviation and improve safety near critical anatomy [51]. However, many of these discussions remain aspirational. They frequently combine evidence from navigation, robotic guidance, and AI planning without clearly separating what each component contributes. This makes it difficult to determine whether the observed gains are driven specifically by AI or by more mature tracking and registration procedures. A more tangible direction is AI-assisted adaptation to motion and environmental disturbance. Vision-guided robotic platforms have been proposed that combine a collaborative robotic arm with three-dimensional camera-based navigation and torque sensing, enabling supervised autonomous drilling and tapping while monitoring forces in real time [30]. This represents a shift toward closed-loop control, where the system responds to measured conditions rather than only enforcing a preplanned geometry. Related work has examined real-time optical-robotic motion compensation as a technical foundation for motion-adaptive implant surgery, particularly to address patient movement as a safety threat [26]. These approaches are scientifically credible and clinically relevant, but most supporting evidence remains experimental or simulated. The broader implant robotics literature also suggests that practical sensing problems still constrain the field. Visual occlusion, marker instability, and changes in instrument geometry can disrupt tracking. Dual-arm concepts have been proposed to reduce occlusion and improve system awareness, underscoring that perception remains a bottleneck even before AI-driven autonomy becomes realistic. In this context, AI should be viewed less as a replacement for robust engineering and more as a means of strengthening weak links, such as registration verification, anomaly detection, and recovery after tracking disruption. A further limitation is methodological. Many AI studies in this space are not designed to support clinical inference. They often lack external validation, rely on retrospective datasets, and report performance metrics that do not map directly onto clinical risk [49]. Even when AI improves accuracy in controlled settings, the clinical value depends on whether it reduces complications, improves functional outcomes, or shortens treatment time without introducing new safety hazards. That evidence is still limited. As robotics begins to incorporate AI for segmentation, planning, and anomaly detection, ethical deployment must be treated as a safety domain, particularly with respect to data privacy, algorithmic bias, and the risk of overtreatment [52]. AI also introduces new failure modes. Errors may be less visible when planning steps are automated, and black-box outputs can create overconfidence in an apparently precise plan. This matters in robotics because robotic execution can faithfully reproduce the plan even when the plan is wrong. The most defensible near-term role for AI in dental robotics is therefore supportive rather than autonomous. It can enhance segmentation, improve registration checks, flag inconsistency, and contribute to motion-aware guidance. Claims about autonomous chairside decision-making should be approached with caution until stronger clinical comparative evidence is available.

Safety, quality assurance, and failure modes

Safety in dental robotics is sometimes discussed as a direct consequence of higher accuracy. That link is plausible, but it is not automatic. Most studies quantify geometric deviation from a planned position. They do not directly measure prevention of harm. The safety profile of robotics is therefore best understood through system failure modes, error propagation, and the quality checks embedded in the workflow. Across robotic and navigation-guided implant surgery, performance depends on a chain of steps. Small errors accumulate across imaging, planning, registration, tracking, and execution. Reviews of robot-assisted implant surgery repeatedly emphasize these workflow dependencies, even when they highlight accuracy advantages [12]. A second challenge is reporting. Many clinical papers report final deviations but provide limited information on intraoperative interruptions, re-registration events, or tracking loss. This restricts interpretability. It also complicates comparisons across systems and centers. The problem is not only academic. These hidden workflow events are often the true safety-relevant signals. The IDEAL Robotics framework is useful here. It treats surgical robotics as complex interventions that require staged evaluation and long-term monitoring. It explicitly highlights human factors, economics, ethics, and real-world surveillance as core components of safe adoption [11].

Sources of Error in Robotic Dentistry

The dominant error sources in dental robotics are not unique to robots. They are errors of spatial truth. The robot executes what it believes is correct. The central question is whether the robot’s coordinate system matches the patient's at the moment of drilling. A primary source of error is registration. If patient-to-plan registration is inaccurate, a robot can reproduce the planned trajectory precisely in the wrong location. Dynamic navigation shows systematic differences between registration methods, with marker-based approaches typically producing better linear accuracy than marker-free alternatives, albeit with workflow trade-offs [32]. These findings generalize to robotics because both modalities rely on the same alignment problem. Tracking instability is another recurrent failure mode. Optical tracking depends on line of sight and stable reference markers.

Dentistry is vulnerable to occlusion because hands, suction, retractors, and the handpiece routinely enter the tracking field. Tracking loss is therefore not an edge case. It is a predictable clinical event that must be managed safely through pauses and re-verification, yet it is often underreported in outcome papers. Technical reviews of implant robotics highlight the narrow workspace and the risk of obstruction as central challenges for reliable positioning [14]. Calibration and mechanical compliance contribute to a different type of error. These errors can be small but systematic. They may present as consistent offsets rather than random noise. This becomes important when studies report mean deviations without examining drift patterns or repeatability across sessions. A retrospective study that aimed to identify factors influencing robotic implant accuracy reflects a broader shift in the literature toward recognizing that accuracy is shaped by multiple workflow and system parameters, not only the robot arm [53]. Also, there is the problem of outcome attribution. A review concludes that robotic systems can achieve high accuracy and stability, especially in controlled settings [54]. Yet deviation metrics alone do not identify where the error entered the pipeline. Without reporting registration quality checks, tracking interruptions, and recovery steps, robot accuracy remains a composite endpoint rather than a mechanistic explanation.

Verification Strategies and Intraoperative Validation

Verification is the mechanism that converts a robotic workflow from technically plausible to clinically defensible. It is also the stage where most preventable failures can be detected early. The aim is not to prove that the robot is theoretically accurate. The aim is to confirm that the robot’s coordinate system still aligns with the patient's at the moment of drilling. In most robotic implant workflows, intraoperative validation begins immediately after registration. The clinician must confirm that the reference markers are stable and that tracking is coherent. If this step is weak, the system can guide drilling confidently while being spatially wrong. This risk is repeatedly acknowledged in implant robotics reviews, which describe registration and verification as complex but essential steps that also increase procedure time. Recent clinical work has tried to reduce variability by defining standardized operating procedures. A study evaluated robotic computer-aided implant surgery in partially edentulous patients under a standard operating procedure and then quantified deviations by superimposing preoperative and postoperative CBCT images [28]. This approach is valuable because it treats workflow control as part of the intervention. It also indirectly shows how fragile the chain can be. Accuracy is not a fixed property of a machine. It is an output of disciplined execution across imaging, registration, tracking, and guidance. Verification becomes even more important during immediate implant placement and anatomically constrained situations. A clinical investigation of robotic computer-assisted surgery for immediate placement highlights accuracy assessment as a primary endpoint, reflecting a broader tendency in the field to use geometric agreement as a proxy for safety. The limitation is that most studies still report deviations without formally documenting intraoperative disruptions, such as re-registration episodes or tracking pauses. Without that data, safe execution is difficult to interpret across centers.

Postoperative Verification, Audit, and Measurement Bias

Postoperative CBCT superimposition remains the most common verification method in robotics and navigation research. It supports the direct quantification of entry deviation, apex deviation, and angular deviation, which are now the most widely used accuracy metrics in computer-assisted implant surgery studies. These outcomes are useful because they are consistent and measurable. They also align well with how many safety claims are framed. At the same time, reliance on CBCT introduces a verification paradox. The imaging modality used as the audit tool is also part of the error chain that created the plan. Voxel size, artifacts, and segmentation uncertainty affect both planning and measurement. This makes postoperative verification vulnerable to shared-method error. To reduce radiation burden and improve practicality, alternative verification methods have been proposed. A prospective cohort study compared a digital registration method using postoperative intraoral scanning with an implant scan body against CBCT-based evaluation for assessing implant positioning [55]. This direction is clinically relevant, but it remains immature. The evidence is not yet strong enough to treat scan-based verification as a universal replacement for CBCT, particularly in complex anatomy or when scan capture is compromised. The broader implication is that verification strategies should be evaluated not only by convenience but also by measurement validity. A method that is easier to apply but more biased can produce misleading performance signals, especially when differences between systems are small.

Failure Modes, Error Propagation, and Recovery Behavior

Safety failures in dental robotics are often framed as rare events. Many are predictable and structural. They arise because the system depends on spatial alignment and uninterrupted tracking. The most common failure types, therefore, are not robot malfunctions. There are mismatches between the virtual plan and the real patient. Registration error is the most consequential failure mode. It does not need to be large to be clinically important. A small misalignment can translate into a clinically meaningful apical shift, especially in narrow alveolar ridges or near neurovascular structures. Tracking problems then amplify this risk. Optical line-of-sight interruptions, marker instability, or patient motion can degrade guidance quality, sometimes without dramatic warning signals. Recent comparative discussions of robotic versus other computer-assisted systems explicitly note that robotic accuracy is still affected by CBCT error, registration error, positioning error, and marker-related issues [8]. A second category is control instability and unintended constraint behavior. Shared-control systems can create a false sense of security if the operator assumes that resistance equals safety. If the spatial map is wrong, constraint forces may prevent correction rather than prevent harm. This is one reason why verification must be continuous, not a single pre-drilling step. Robotic workflows require explicit recovery behavior, including when to pause, re-register, or convert to freehand. Implant robotics reviews frequently emphasize that these workflows operate under human oversight and that the surgeon remains central to execution decisions. The literature also shows that performance can improve when workflow is standardized. That is reassuring, but it also means outcomes are process-dependent. The robot is not a guarantee. It is a precision tool embedded in a vulnerable chain [28].

Training, Credentialing, and Long-Term Safety Monitoring

Training is a safety intervention. It determines whether robotics reduces error or merely shifts it to earlier steps, such as registration and verification. In dentistry, this issue is increasingly visible because robotic workflows redistribute cognitive load. Manual dexterity becomes less dominant, while system discipline becomes more important. Dental evidence is now emerging on learning curves in robot-assisted implant surgery. Studies examining task-autonomous systems in younger clinicians and trainees reflect the field’s recognition that competence must be developed and measured, rather than assumed [46]. This aligns with earlier learning-curve research in dynamic navigation, which showed that accuracy and efficiency can change markedly during early training phases. The implication for robotics is straightforward. Reported accuracy in expert hands should not be generalized to routine adoption without structured training and verification standards. The most rigorous governance model currently comes from outside dentistry. The IDEAL Robotics framework argues that robotic technologies should be evaluated through a staged development process, emphasizing comparative studies and long-term monitoring based on transparent real-world data collection [11]. This matters because many dental robotics claims rely on small studies, selected cases, and short follow-up. Long-term surveillance would allow detection of rare complications, performance drift, and center-level variability. A recent qualitative study on robotic surgery training needs, although not dental-specific, reinforces that training design is often inconsistent and that formal curricula are still evolving [11]. Dentistry is likely to face the same structural problem. Without standardized credentialing, robot-assisted surgery can become a label with unpredictable clinical meaning (Table 2).

**Table 2:** Clinical takeaways on dental robotics across outcomes, safety, and value domains Table content synthesized from the reviewed literature [7,8,11-14,25-30,46-48,54].

Clinical outcome	Main findings	What appears consistent across studies	What remains uncertain or limited	What this means for practice now
Positional accuracy	Robotic systems tend to deliver strong agreement between the planned and achieved implant position.	Accuracy gains are most visible for angulation and apical positioning, particularly when strict restorative-driven placement is required.	Accuracy is still constrained by imaging quality, registration fidelity, and tracking stability, so performance is not uniform across workflows.	Robotics is most defensible when precision has direct clinical consequences, such as limited bone volume, narrow safety margins, or demanding prosthetic trajectories.
Accuracy compared with freehand, static guides, and navigation	The advantage over freehand is usually clearer than the advantage over other guided modalities.	Robotic workflows reduce operator-dependent variation and tend to narrow the spread of placement deviations.	Differences between robotic and other guided approaches can be modest and highly dependent on case complexity, operator experience, and the quality of digital planning.	Robotics should be positioned as an alternative within computer-assisted surgery, not as a universal replacement for static or dynamic guidance.
Procedural efficiency and operative time	Robotics often improves control during drilling but adds workflow steps before drilling begins.	Early cases tend to take longer, mainly due to registration, calibration checks, and workflow interruptions.	Time outcomes are difficult to compare because studies define procedure time differently and rarely separate setup from execution.	Time savings should not be the primary argument for adoption. Efficiency may improve after standardization and team familiarity.
Intraoperative safety events and technical disruptions	The main safety threats are workflow-related rather than mechanical failure of the robot.	Tracking interruption, reference instability, and the need for re-registration are recurring real-world events.	These events are often underreported, which limits understanding of how frequently workflows need rescue or conversion.	Safe use depends on disciplined verification routines and clear stop rules. A conversion-to-manual pathway should be part of routine planning.
Postoperative complications	Complication rates appear broadly comparable to those of conventional implant surgery across the studied indications.	Most reports describe acceptable short-term healing and similar postoperative morbidity profiles.	Many studies are not powered to detect uncommon complications, and follow-up is often short.	Robotics can be presented as clinically safe within selected cases, but superiority for preventing harm should be stated cautiously.
Implant survival and success	Short-term survival is typically high and does not differentiate robotics clearly from established approaches.	Early survival tends to be excellent across techniques, so it is rarely a sensitive endpoint for comparison.	Long-term outcomes remain insufficient to conclude that higher placement precision improves survival, peri-implant stability, or the maintenance burden.	Claims should focus on placement fidelity and workflow benefits rather than long-term survival advantages until longer follow-up becomes routine.
Peri-implant hard and soft tissue outcomes	Tissue outcomes may benefit from improved robotics in prosthetic positioning and emergence design, but evidence remains indirect.	Radiographic and soft-tissue measures are usually stable during short follow-up, and overt harm signals are uncommon.	Tissue health depends strongly on prosthetic contours, hygiene access, and maintenance, which are not always controlled in comparisons.	Robotics may support tissue preservation in high-demand esthetic or contour-sensitive cases, but it is not a substitute for prosthetic and maintenance quality.
Prosthetic and restorative outcomes	The most plausible downstream benefit is smoother restorative delivery when the planned prosthetic geometry is reliably achieved.	Reports often imply fewer positional surprises at the restorative stage and fewer compensatory prosthetic adjustments.	Hard prosthetic endpoints are inconsistently measured, and laboratory workflow effects are rarely isolated from surgical effects.	Restorative-driven clinics may see the clearest practical gains, especially when a consistent implant axis and emergence are critical for the final design.
Patient-reported outcomes and experience	Patient experience does not appear uniformly better, but it is generally acceptable and may improve postoperatively in some contexts.	Satisfaction is often high, and patients tend to accept robotic procedures when the benefits are explained clearly.	PROMs vary in timing and instruments, and patient perception may be influenced by novelty and expectations.	Patient communication should emphasize precision, support, and safety checks, rather than “automation” or “robot does the surgery,” to avoid misleading impressions.
Learning curve and operator variability	Robotics shifts the competence profile from manual control to workflow control.	Accuracy can be maintained across operators once the workflow is mastered, thereby supporting standardization efforts.	Learning curves are not fully characterized across settings, and performance from expert centers may not reflect typical adoption.	Training should prioritize registration discipline, verification behavior, and failure recovery, not only drilling execution.
Cost, access, and service value	Cost remains the strongest barrier to widespread adoption, and value is indication-dependent.	Higher equipment and maintenance costs are consistently described, alongside operatory footprint and training demands.	Formal cost-effectiveness work remains limited, and savings from avoided complications are difficult to demonstrate.	Robotics is best positioned as a selective service for cases where precision adds clear clinical or restorative value.
Overall certainty of evidence and research priorities	The field shows promising performance signals, but comparative certainty remains moderate at best.	Accuracy improvement is the most stable finding; feasibility and safety are supported within the studied indications.	Evidence is limited by heterogeneous protocols, selective case inclusion, short follow-up, and incomplete reporting of disruptions and conversions.	Future work should prioritize pragmatic comparisons, standardized outcomes, transparent reporting of failures, and longer-term clinical endpoints.

## Conclusions

Dental robotics is moving from proof of concept to a practical adjunct in digitally planned dentistry. Current evidence suggests that shared-control, robot-assisted implant workflows can achieve high agreement between planned and achieved implant positions, with the clearest accuracy gains versus freehand placement, while also introducing a real implementation trade-off. Beyond implantology, robotics is already established in orthodontic archwire bending and in selected oral and maxillofacial applications, but these domains follow different evidence pathways. At the same time, the evidence base remains heterogeneous, with an overemphasis on geometric accuracy outcomes and less consistent reporting of complications, patient-reported outcome measures, long-term maintenance, learning curves, workflow disruptions, and economic value. Future research should prioritize pragmatic comparative studies with standardized outcomes, transparent reporting of failures and interruptions, longer follow-up, and formal economic evaluation.
